# Proceedings at the Annual Convention of the Connecticut Medical Society, May, 1848, Etc.

**Published:** 1848-08

**Authors:** 


*Proceedings at the Annual Convention of the Connecticut Medical Society, May*, 1848, *etc.*

We are indebted to the politeness of Dr. Gordon W. Russell, secretary of the Connecticut Medical Society, for a copy of the proceedings at their last annual convention. The reader of this publication cannot fail to entertain sentiments of great respect for the intelligence and spirit which appear to pervade the Medical Profession of Connecticut. In connection with the proceedings, is published an address by B. Fordyce Baker, M. D., on *Diseases of the Cervix Uteri*, which we have read with pleasure and profit. We had marked the address for copious quotations, but have concluded not to insert them, as the subject has been considered pretty fully in a former number of this Journal (see analytical Review of Bennett on diseases of uterus, vol. 11 page 575) Dr. Baker's observations go to confirm the views contained in the article just referred to, and illustrate the importance of the speculum vaginæ, in determining the true morbid conditions of the cervix, and in applying remedies directly to the diseased parts. We commend Dr. Baker's address to the careful perusal of the practitioner.

